# Human Serum Albumin Affinity for Putrescine Using ITC and STD-NMR

**DOI:** 10.3390/ijms26136084

**Published:** 2025-06-25

**Authors:** Vida Dehghan Niestanak, Ryan McKay, Marcello Tonelli, Larry D. Unsworth

**Affiliations:** 1 Department of Biomedical Engineering, University of Alberta, Edmonton, AB T6G 2V4, Canada; vidadehghan@ualberta.ca; 2 Department of Chemistry, University of Alberta, Edmonton, AB T6G 2V4, Canada; ryan.mckay@ualberta.ca; 3 Department of Medicine, University of Calgary, Calgary, AB T2N 2T8, Canada; cello@ucalgary.ca; 4 Department of Chemical and Materials Engineering, University of Alberta, Edmonton, AB T6G 2V4, Canada

**Keywords:** protein-bound uremic toxins, putrescine, human serum albumin, isothermal titration calorimetry, STD NMR

## Abstract

Understanding the binding interactions between protein-bound uremic toxins (PBUTs) and human serum albumin (HSA) is critical for advancing treatments for chronic kidney disease (CKD). While previous studies have suggested that putrescine, a diamine PBUT, exhibits moderate binding affinity to HSA, this study provides evidence of the contrary. Using isothermal titration calorimetry and saturation transfer difference nuclear magnetic resonance , we demonstrate that putrescine’s interaction with HSA is weak, non-specific, and thermodynamically negligible in the range of conditions studied. Unlike earlier studies relying on spectroscopy techniques such as UV–visible absorption and fluorescence, which may overestimate binding strength, the results presented here highlight the limitations of indirect methodologies and underscore the importance of more sensitive approaches for accurate energy characterization. Our findings suggest that putrescine only weakly interacts non-specifically with HSA and may bind more preferentially to other plasma proteins, contributing to its accumulation in CKD patients.

## 1. Introduction

Kidney failure leads to the accumulation of uremic metabolites in the blood compartment. While hemodialysis (HD) has proven to be a life-saving treatment for patients with kidney failure, persistently high mortality rates and comorbidities like cardiovascular disease as well as oxidative stress and inflammation are too common in this patient population [1,2,3]. HD does not clear all uremic metabolites equally, and those characterized by a high affinity for plasma proteins are almost impossible to clear using conventional methods [3,4,5]. Understanding the binding interactions between protein-bound uremic toxins (PBUTs) and plasma proteins is essential for designing effective adsorbents and competitive displacers to enhance conventional treatments for patients with End-Stage Renal Disease (ESRD)) [3,6]. The average concentrations of putrescine are 10.13 and 59.03 μg/L in people with normal kidney function and ESRD patients undergoing dialysis, respectively [7], which highlights the accumulation of this uremic metabolite despite the receipt of HD treatment. This study focuses on the binding mechanism of putrescine, a recognized PBUT, to human serum albumin (HSA).

HSA accounts for 50% to 60% of human plasma proteins, with a concentration of approximately 40 g/L. Under physiological conditions, it carries a net negative charge of −19 mV and has an isoelectric point of 5.4 [8]. HSA is composed of 585 amino acids organized into three homologous α-helical domains (I, II, and III), each containing around 10 helices, which form two long loops and one shorter loop. The first two loops constitute subdomain A within each domain, while the shorter loop forms subdomain B. These subdomains exhibit distinct ligand-binding properties and functional roles despite their structural similarities [9]. As the most abundant protein in human plasma, HSA plays a critical role in modulating the pharmacokinetics of various drugs and serves as a primary binding site for endogenous metabolites [3,10]. Research has identified Sudlow sites I and II, situated in subdomains IIA and IIIA, respectively, as the primary binding sites for PBUTs on HSA [3]. These sites also serve as key binding locations for bulky heterocyclic and aromatic compounds, such as warfarin and ibuprofen, which exhibit strong interactions with sites I and II, respectively [11,12].

Putrescine, a diamine, plays a critical role as a metabolic precursor in synthesizing polyamines such as spermidine and spermine, which are essential for cell proliferation and act as growth factors for cell division [13]. As noted earlier, putrescine levels are significantly elevated in patients with kidney disease [14]. Dialysis is not very effective for removing putrescine from plasma, suggesting that its concentration could contribute to the high rates of adverse outcomes among people with ESRD [15]. This elevation is part of the broader disruptions in polyamine metabolism observed in kidney disease, with potential implications for cellular processes and disease progression [15,16]. Interestingly, while putrescine levels increase in ESRD patients, the plasma concentrations of other polyamines, such as spermidine and spermine, often decrease [16]. However, UV–visible absorption spectroscopy measurements indicate that putrescine exhibits a slightly lower binding affinity for human serum albumin (HSA) than spermine and spermidine [17].

Previous studies have reported that putrescine can bind approximately 15 different plasma proteins [18]. The major findings predominantly relied on spectroscopy techniques, including UV–visible absorption spectroscopy, fluorescence spectroscopy, Fourier-transform infrared (FTIR) spectroscopy, and circular dichroism (CD) [19,20]. These methods have indicated that the association constant of putrescine with HSA and bovine serum albumin (BSA) is approximately 10^3^ M^−1^ in pH ~ 7.4 at 25 °C [20,21]. Similarly, studies have shown that putrescine interacts with bovine trypsin in an exothermic manner, accompanied by negative entropy changes, and exhibits a relatively higher association constant (~10^6^ M^−1^) [19]. The limitations of the spectroscopic techniques commonly used in prior studies are worth noting. For instance, fluorescence spectroscopy, while highly sensitive to changes in the nano-environment of fluorophores, can lead to misinterpretations if experimental conditions are not carefully controlled [22,23]. Furthermore, these methods often cannot differentiate between direct ligand binding, allosteric changes, or ligand-induced interactions. They may not always accurately represent the accurate thermodynamic parameters governing interaction.

Building on this foundation, our study investigates the binding interactions of putrescine with HSA using advanced techniques such as isothermal titration calorimetry (ITC) and saturation transfer difference nuclear magnetic resonance (STD-NMR). These methods reveal minimal interaction between putrescine and HSA, offering a more nuanced perspective on the possibility of putrescine exhibiting higher affinity for other plasma proteins, potentially cysteine-rich proteins [24]. The results presented here highlight the limitations of indirect methods for determining binding characteristics by showing that very little interaction occurs between putrescine and HSA.

## 2. Results and Discussion

### 2.1. Putrescine Binding to HSA

**Figure 1:**
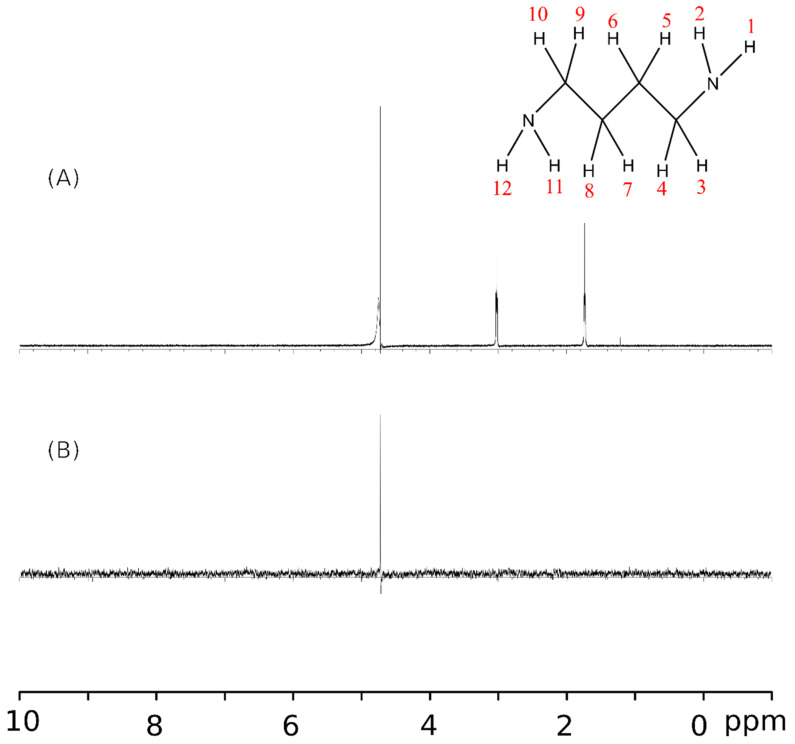
1D-^1^H NMR of putrescine (**A**) with chemical shifts ~1.5–2 ppm (H5, H6, H7, H8) and ~2.9–3.1 (H3, H4, H9, H10) [13] and STD-NMR spectra of putrescine–HSA (**B**). The large shift in the middle is for the reference solution (10% H_2_O present).

STD-NMR selectively enhances the signals of ligand regions very near or in direct contact with the protein while suppressing signals from unbound molecules in solution [25]. A comparison between the 1D-^1^H NMR spectrum of free putrescine (Figure 1A) and the STD-NMR spectrum of putrescine in the presence of HSA (Figure 1B) revealed that no protons on putrescine are near the protein, indicating the absence of magnetization transfer between the molecules and suggesting that putrescine does not exhibit specific or close interactions with HSA. Although putrescine may still interact with HSA, the lack of expected signal enhancement suggests that any interaction is weak at best or non-existent at worst. If any binding occurs, it is likely in rapid exchange compared to the NMR timescale, where putrescine molecules associate and dissociate from HSA too quickly to produce detectable signals in the STD-NMR experiment.

### 2.2. Interaction Thermodynamics

**Figure 2:**
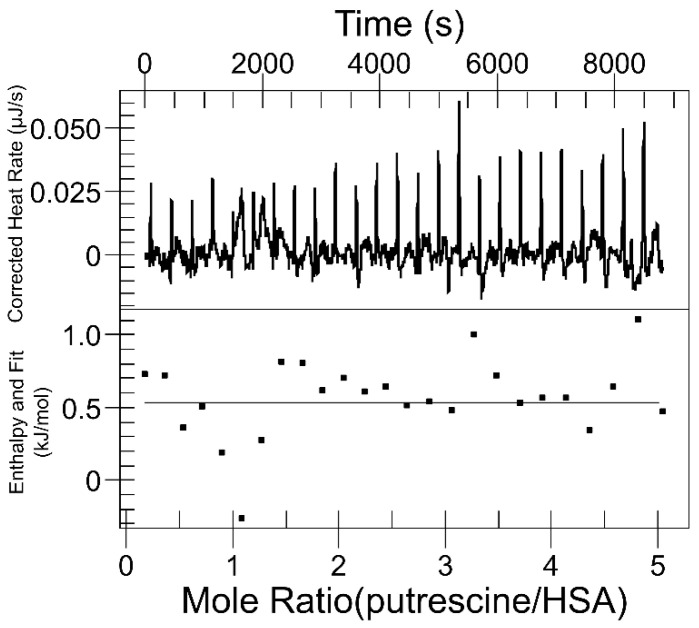
ITC baseline-corrected thermograms (**upper panel**) and the fitting of multiple binding sites (**lower panel**). Putrescine with an initial concentration of 1.5 mM was injected to 0.1 mM HSA at 298 K and pH = 7.40. Baseline correction was conducted by subtracting the thermogram of putrescine injections into the buffer to account for the dilution heat.

To further investigate the binding mechanisms between putrescine and HSA, isothermal titration calorimetry (ITC) experiments were conducted by titrating a 1.5 mM putrescine solution into 0.1 mM HSA (Figure 2) and modeled with manufacturer-provided tools. The results showed consistent low-intensity heat peaks with no observable saturation with injection number while titrating 0.1, 0.5, 1, and 1.5 mM putrescine, indicating that the heat changes in each injection were minimal and nearly indistinguishable from baseline noise. This consistent heat area suggests a weak interaction between putrescine and HSA, with the observed heat changes likely arising from non-specific interactions, with no evidence of strong interactions or significant conformational changes in the protein.

These findings contrast previous studies conducted under similar environmental conditions (pH = 7.2–7.4; T = 25 °C). One study, using UV-vis absorption spectroscopy, reported an interaction between 1 mM putrescine and 1.5 mM HSA with a K_a_ ~ 10^3^ [20]. Another study on putrescine–bovine serum albumin (BSA) at pH 7.4 and 25 °C, using protein fluorescence quenching (0.001 mM BSA with 0–0.4 mM putrescine), yielded a K_a_ ~ 0.6 × 10^2^ [21]. The discrepancy in K_a_ values between studies could be partially attributed to differences in protein concentration. Higher protein concentrations might enhance weak interactions, potentially explaining the higher K_a_ observed in the first study. However, this does not account for the similar K_a_ reported in the putrescine–BSA study, where the protein concentration was 1000-fold lower. This inconsistency underscores the complexity of interpreting binding data across different experimental setups. Another key methodological difference between our study and previous reports includes using phosphate buffer instead of Tris-HCl. While buffer composition and protein concentration can influence binding measurements, a strong and specific interaction should generally be detectable across different experimental conditions. The minimal interaction observed in our ITC and STD-NMR results suggests that putrescine’s affinity for HSA is inherently weak rather than an artifact of buffer or concentration differences. However, it is possible that the putrescine–HSA interaction is highly sensitive to experimental conditions, including buffer composition and concentration ratios. Future studies could systematically compare these conditions to assess their impact on interaction.

ITC and STD-NMR, employed in this study, are more sensitive and direct methods for measuring binding [11]. These advanced techniques provide a clearer picture, often revealing weak or non-specific interactions that earlier methods might misinterpret as significant binding events. The absence of detectable interaction in our ITC and STD-NMR experiments strongly suggests that the putrescine–HSA interaction is weak or non-existent under our conditions. UV–visible absorption and fluorescence spectroscopy have limitations that could lead to the overestimation of binding strength. For example, fluorescence spectroscopy relies on changes in the environment of tryptophan residues, particularly Trp214 in HSA [26], which may not accurately reflect the overall binding interaction, especially if putrescine interacts at sites distant from the fluorophores. Such techniques can detect non-specific interactions or minor conformational changes, which may not correspond to actual, strong binding events [22]. In contrast, the negligible energetics observed in this study underscore the absence of significant binding between putrescine and HSA.

## 3. Methods and Materials

### 3.1. Materials

Human serum albumin (HSA, purity ≥ 98%, fatty acid-free, 66.4 kDa) and putrescine dihydrochloride (purity ≥ 98%, powder, 161.07 g/mol) were purchased from MilliporeSigma Company (USA). Protein solutions were freshly prepared for each experiment, and their concentrations were verified by measuring absorbance at 280 nm using a Nanodrop spectrophotometer (ThermoFisher, Waltham, MA, USA) with a UV–vis detector and a molar absorption coefficient of 35,700. Buffer compositions varied based on the requirements of each instrument, as detailed in the subsequent sections. All buffers were prepared at room temperature (25 °C) with their pH adjusted to 7.4 and verified using an EcoSence^®^ model pH10A pH meter (YSI Incorporated, Yellow Springs, OH, USA). Buffer stock solutions were stored at 4 °C and filtered through 0.2 µm syringe filters prior to use.

Phosphate-buffered saline (PBS) tablets, obtained from Fisher Scientific (MilliporeSigma Canada Ltd., Oakville, ON, Canada), were used to prepare a 0.01 M phosphate buffer containing disodium phosphate and potassium dihydrogen phosphate, 0.0027 M potassium chloride, and 0.137 M sodium chloride. For NMR experiments, 99.9% D_2_O (100 g) was purchased from Sigma-Aldrich (Oakville, ON, Canada); although it was opened and used across multiple projects, the exact concentration of D_2_O was not specified. NMR tubes (part number 502-7) were procured from Norell (Morganton, NC, USA).

### 3.2. Saturation Transfer Difference NMR

All samples were prepared in PBS solutions made with distilled and deionized H_2_O and D_2_O (90% *v*/*v* D_2_O). Each 5 mm NMR tube was loaded with 700 μL of prepared solutions. Sample components were stored on ice during transport to the NMR facility, and experiments were conducted immediately after sample preparation to ensure accuracy.

All saturation difference NMR experiments [25,27,28,29,30] and associated sample 1D-^1^H experiments were conducted on a 14.1 T (600 MHz) Varian/Agilent VNMRS system operating at 27 °C calibrated to methanol [31]. The 600 MHz spectrometer utilized a 5 mm autoxdb direct-detect ^1^H inner coil. Preliminary proof of concept and concentration confirmation data were acquired on a 400 MHz Inova spectrometer. All spectra were acquired under lock conditions using the internal ^2^H resonance, with chemical shifts referenced to the residual ^1^HOD signal at 4.7 ppm before saturation [32,33,34].

One-dimensional ^1^H spectra were collected using presaturation (2 s at ~85 Hz gammaB_1_ induced field) of the residual water resonance [35,36] followed by a calibrated single 90° hard excitation pulse and 3 s of acquisition time. The saturation pulse and carrier position were manually optimized to align with the water resonance. Shimming and water suppression were optimized to prevent ADC/receiver overloads. Acquisition parameters included a sweep width of 7183 Hz (~12 ppm) and 43,104 total real and imaginary points. Automatic tune/match and gradient shimming were used on each sample.

Saturation transfer difference spectra (Supplementary document, Figure S1) (i.e., Varian/Agilent ‘std1d’ pulse sequence) were acquired utilizing a 50 ms eburp1 [37] selective pulse (95 Hz gammaB_1_) repeated 20 times with an internal 1 ms recovery delay between pulses. As per the well-established standard protocols of saturation transfer experiments [25,27,28,30,38,39,40,41,42], the selective pulse was positioned either at −1 ppm (on-resonance) or +40 ppm (off-resonance), and the difference spectra were collected via the receiver’s internal phase cycling. Another version of the pulse sequence using an on-resonance pulse at ~3 ppm was used to confirm results. However, the on-resonance pulse at 3 ppm indicated some slight possible cross-excitation into particular ligands and for simplicity, only the traditional pulse sequence data was shown and utilized for calculations. The pulse sequence also contained the improved watergate suppression W5 method [43,44,45] with 400 μs internal delays. A 30 ms “spinlock” (2394 Hz gammaB_1_) was used before the W5 for significant molecule/protein signal suppression. Pulsed-field gradients (~24 g/cm, 1 ms duration) were used at either end of the suppression component. Total acquisition time was 2 s with 28,746 points acquired (sweep width also 7183 Hz). A total of 128 scans were acquired for a total experiment time of ~7 min.

NMR data processing included zero-filling to double the acquired points, applying a 1 Hz line-broadening apodization function, and manual phasing. No baseline correction was performed. Chemical shifts were confirmed via the solvent peak based on the lock solvent and carrier position. Spectra were analyzed using OpenVnmrJ VERSION 3.2 (current release 6 September 2024, available from https://openvnmrj.org, accessed on 14 May 2025).

### 3.3. Isothermal Titration Calorimetry

Isothermal titration calorimetry (ITC) experiments were conducted using a Nano-ITC instrument (TA Instruments, New Castle, DE, USA). Samples were prepared in PBS buffer (0.01 M, pH 7.4) and degassed for 15 min before use. Thermal equilibrium was achieved through two stabilization processes (each lasting approximately 1 h), ensuring that heat transfer fluctuations between the sample and reference cells remained below 0.02 μJ/s. After stabilization, a syringe containing 50 μL of ligand solution was titrated into the protein sample cell, which held 170 μL of solution. A ligand-to-HSA molar ratio of 15 was maintained in all experiments (0.1 mM HSA and 1.5 mM putrescine). Each experiment consisted of 25 injections (2 μL each), with raw heat data recorded for each injection over time. Experiments were performed in triplicate, and the standard deviations of the isotherm parameters are reported.

The cumulative heat from each injection was translated into molar heat change as a function of the molar ratio of titrant to protein. Thermodynamic parameters, including enthalpy change (ΔH), entropy change (ΔS), Gibbs free energy change (ΔG), and the association binding constant (K_a_), were calculated at 25 °C using standard thermodynamic equations (Equations (1) and (2)). Here, R represents the universal gas constant, and T is the temperature (K) [46]. The model incorporated accounted for potential binding sites, as described previously [3,11].

(1) ∆G=∆H−T∆S

(2) $$∆G=-RTln(K_{a})$$

## 4. Conclusions

This study explores the binding interaction between putrescine and HSA, challenging previous reports of moderate affinity. We use advanced analytical techniques like ITC and STD-NMR to reveal that putrescine’s interaction with HSA is too weak to determine using ITC and STD-NMR techniques for these conditions. The negligible thermodynamic changes observed during ITC experiments and the absence of detectable binding via STD-NMR indicate that putrescine does not form strong or specific interactions with HSA. These findings highlight the limitations of conventional spectroscopy methods, which may misinterpret weak or indirect interactions as strong binding. Furthermore, the weak interaction between putrescine and HSA suggests that the accumulation of putrescine in ESRD patients may not be due to its affinity with HSA but to its interactions with other plasma proteins or metabolic imbalances in polyamine pathways. This study contributes to the broader understanding of PBUT interactions with plasma proteins by providing a more accurate characterization of putrescine’s binding behavior. These insights are essential for designing effective adsorbents and competitive displacers to enhance treatments for ESRD and emphasize the need for more precise and sensitive methodologies in future research.

## Data Availability

All the data generated in this research has been provided in the manuscript, and any further information will be available upon request.

## Supplementary Materials

The following supporting information can be downloaded at https://www.mdpi.com/article/10.3390/ijms26136084/s1.
