# Supplementary material for: Human Serum Albumin Affinity for Putrescine Using ITC and STD-NMR
Source: Int J Mol Sci. 2025 Jun 25;26(13):6084. doi: 10.3390/ijms26136084 (PMC12249803; doi:10.3390/ijms26136084)
Supplement: Supplementary file 1 [file ijms-26-06084-s001.zip › ijms-3672412-supplementary.pdf]

Tryptophan-Albumin Interaction a Potential Protein-Bound Uremic Metabolite

Vida Dehghan Niestanak, MSc

Department of Biomedical Engineering, University of Alberta, Edmonton, Canada

vidadehghan@ualberta.ca

Ryan M[c]Kay, PhD

Department of Chemistry, University of Alberta, Edmonton, AB, Canada

ryan.mckay@ualberta.ca

Marcello Tonelli, MD, SM, MSc

Department of Medicine, University of Calgary, Calgary, Canada

cello@ucalgary.ca

Larry D. Unsworth, PhD, PEng

Department of Chemical and Materials Engineering, University of Alberta, Edmonton, Canada

lunswort@ualberta.ca

Correspondence to:

Larry D. Unsworth, University of Alberta

DICE 13-390

University of Alberta

Edmonton, AB

T6G 2V4

Ph: 780-492-6020

Fax: 780-492-2881

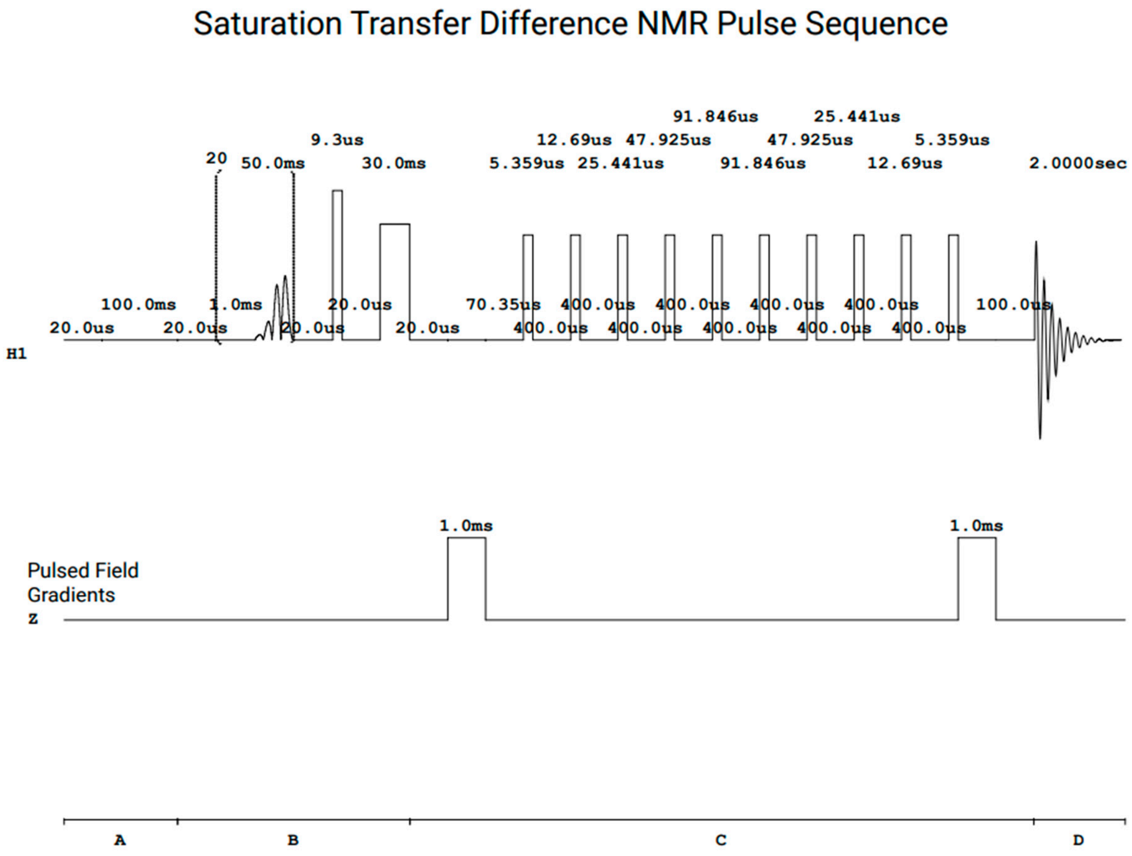

Figure S1 – Saturation transfer difference NMR pulse sequence
